# Serotype-Specific Killing of Large Cell Carcinoma Cells by Reovirus

**DOI:** 10.3390/v9060140

**Published:** 2017-06-06

**Authors:** Emily J. Simon, Morgan A. Howells, Johnasha D. Stuart, Karl W. Boehme

**Affiliations:** Department of Microbiology and Immunology and Center for Microbial Pathogenesis and Host Inflammatory Response, University of Arkansas for Medical Sciences, Little Rock, AR 72205, USA; esimon@uams.edu (E.J.S.); mahowells@uams.edu (M.A.H.); jdstuart@uams.edu (J.D.S.)

**Keywords:** reovirus, oncolysis, lung cancer, reverse genetics

## Abstract

Reovirus is under development as a therapeutic for numerous types of cancer. In contrast to other oncolytic viruses, the safety and efficacy of reovirus have not been improved through genetic manipulation. Here, we tested the oncolytic capacity of recombinant strains (rs) of prototype reovirus laboratory strains T1L and T3D (rsT1L and rsT3D, respectively) in a panel of non-small cell lung cancer (NSCLC) cell lines. We found that rsT1L was markedly more cytolytic than rsT3D in the large cell carcinoma cell lines tested, whereas killing of adenocarcinoma cell lines was comparable between rsT1L and rsT3D. Importantly, non-recombinant T1L and T3D phenocopied the kinetics and magnitude of cell death induced by recombinant strains. We identified gene segments L2, L3, and M1 as viral determinants of strain-specific differences cell killing of the large cell carcinoma cell lines. Together, these results indicate that recombinant reoviruses recapitulate the cell killing properties of non-recombinant, tissue culture-passaged strains. These studies provide a baseline for the use of reverse genetics with the specific objective of engineering more effective reovirus oncolytics. This work raises the possibility that type 1 reoviruses may have the capacity to serve as more effective oncolytics than type 3 reoviruses in some tumor types.

## 1. Introduction

Oncolytic viruses are an emerging class of cancer therapeutics that selectively replicate in and kill transformed cells while sparing non-transformed cells [1,2]. Mammalian orthoreovirus (reovirus) is one of a number of viruses with oncolytic potential that are in different stages of development [2,3,4,5,6]. Reoviruses are non-enveloped viruses with a segmented double-stranded RNA genome that productively infect and lyse several different tumor cell types in vitro and in experimental animal models [3,4,7]. In some cases, reovirus shows efficacy as a virotherapeutic agent for aggressive and refractory human tumors [8]. Phases I and II clinical trials in the United States, Canada, and Europe demonstrate that pelareorep (Reolysin), a first-generation reovirus therapeutic is tolerated and safe, even in patients with advanced cancer who have undergone extensive chemotherapy [9,10]. A Phase III clinical trial to test reovirus efficacy against head and neck cancers was recently completed.

Phase I and II trials indicate that the majority of oncolytic viruses under development, including reovirus, are safe for use in humans [11]. However, the therapeutic efficacy of many of these agents is limited [2]. The therapeutic potency of oncolytic viruses can be enhanced by genetic modifications that increase viral replication in and killing of cancer cells, re-target the virus to infect new tumor types, or introduce immunomodulatory factors that enhance immune-mediated killing of tumors [2]. For example, talimogene laherparepvec (T-VEC), which in 2015 became the first oncolytic virus approved by the US Food and Drug Administration (FDA) for use in humans, is a genetically-modified herpes simplex virus type 1 (HSV-1) [12]. The HSV-1 strain that forms the basis for T-VEC lacks the viral *ICP34*.5 gene, which enhances tumor cell lysis. *ICP34*.5 also is required for replication in the central nervous system (CNS) and its deletion further improves the safety profile of the virus [13]. The *ICP34*.5 locus was replaced with the gene encoding granulocyte-macrophage colony-stimulating factor (GM-CSF), a cytokine that functions to recruit and activate antigen presenting cells that facilitate T-cell recognition and killing of cancer cells [14]. Other prospective oncolytic viruses, including myxoma virus [15], adenovirus [16], coxsackievirus [17], measles virus [18], and vesicular stomatitis virus [19] also have been genetically altered to increase safety or oncolytic efficacy [2].

In contrast, reovirus has not undergone genetic manipulation to increase its therapeutic potency [2,20]. The reovirus in use for clinical trials (Reolysin) is a derivative of laboratory strain type 3 Dearing (T3D) [4] that was isolated from a child’s stool sample in the 1950s [21]. Although Reolysin shows tremendous efficacy in pre-clinical studies, initial clinical trials showed that the efficacy of reovirus monotherapy was low [22,23]. As a consequence, ongoing clinical trials use Reolysin in combination with chemotherapy or radiotherapy [11]. However, it may be possible to enhance the oncolytic capacity of reovirus, as large plaque variants generated by serial passage of the Reolysin parental strain T3D in L929 cells showed an increased capacity to lyse tumor cells [24]. This finding indicates that reovirus can be further adapted to more effectively kill tumors. A wealth of reovirus sequence polymorphisms that augment viral replication or host responses are known [25]. Some of these polymorphisms may have the capacity to increase reovirus oncolytic activity. The segmented reovirus genome also allows generation of reassortant viruses that contain distinct combinations of gene segments from different reovirus strains. Many reovirus strain-specific differences in properties that may enhance oncolysis, including viral replication, apoptosis induction, and host cell tropism are linked to a single gene segment. Reassortant reoviruses that contain a combination of gene segments from different reovirus strains may enhance viral oncolytic efficacy. In addition, it may be possible to combine individual mutations and reassortants to increase reovirus oncolytic activity or generate reoviruses that are uniquely tailored to target specific tumor types. Cancer is a highly heterogeneous disease that can originate from any type of cell in the body [26]. The genetic or epigenetic abnormalities that lead to transformation often differ greatly between individuals that have the same type of cancer [26]. Even within a single tumor, there are often dramatic differences between the cells that comprise the mass [26]. Together, cancer heterogeneity leads to differential susceptibility to anti-cancer therapeutics, including reovirus [27]. The ability to generate reoviruses with different constellations of gene segments that enhance killing in different cell and tumor types could greatly expand the utility of reovirus as a cancer treatment.

Reovirus can be genetically modified using plasmid-based reverse genetics [28,29,30,31]. Complete reverse genetics systems are available for the two most commonly studied prototype reovirus laboratory strains, T3D and type 1 Lang (T1L) [28,29,30]. Although recovery of recombinant reovirus is highly efficient, this technology has not been fully applied to the production or enhancement of reovirus for oncolytic purposes. In this study, we tested recombinant strains (rs) of T1L (rsT1L) and T3D (rsT3D) that were generated by plasmid-based reverse genetics for the capacity to kill a panel of non-small cell lung cancer (NSCLC) cell lines (Table 1). Lung cancer is the leading cause of cancer-related deaths among both men and women in the United States [32]. The 5-year survival rate for lung cancer is approximately 17% [33] and over half of patients succumb within one year of diagnosis [34]. NSCLC causes 85% of lung cancer cases and can be divided into three subtypes NSCLC—adenocarcinoma, large cell carcinoma, and squamous cell carcinoma [35]. Our cell panel consisted of three adenocarcinoma and two large cell carcinoma cell lines. Adenocarcinomas are the most prevalent NSCLC subtype and large cell carcinomas are generally the most aggressive form of the disease [35]. We found that rsT1L and rsT3D killed cell lines derived from lung adenocarcinomas equivalently. However, rsT1L killed the large cell carcinoma cell lines tested to a substantially greater extent than rsT3D. Importantly, recombinant and non-recombinant reoviruses killed each cell line tested equivalently. Production of viral yields by rsT1L and rsT3D were comparable in the large cell carcinoma cell lines. This finding indicates that differences in viral replication do not underlie the strain-specific disparity in killing of the large cell carcinoma cell lines. Finally, we identified reovirus gene segments, L2, L3, and M1 as viral determinants of differential cell killing of large cell carcinoma cell lines. Together, our results indicate that that the oncolytic potency of reoviruses generated by reverse genetics is comparable to non-recombinant strains. Our results further suggest that a T1L-based reovirus may be more effective therapeutics than T3D-derived viruses in certain cell or tumor types.

## 2. Results

### 2.1. Recombinant Reovirus Strains rsT1L and rsT3D Differ in the Capacity to Kill Large Cell Carcinoma Cell Lines

**Figure 1:**
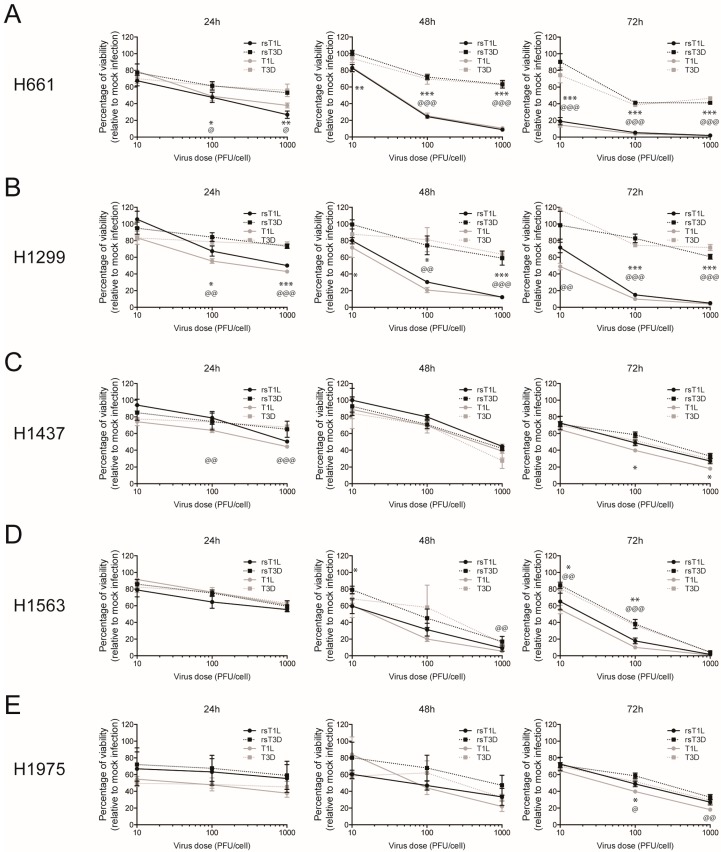
Lung cancer cells are susceptible to killing by recombinant reoviruses. (**A**) H661; (**B**) H1299; (**C**) H1437; (**D**) H1563; and (**E**) H1975 cells were infected with recombinant strains (rs)T1L, rsT3D, T1L or T3D at multiplicity of infection (MOIs) of 10, 100, or 1000 plaque forming units (PFU)/cell. Cellular ATP content was measured at 24 h (left panel), 48 h (middle panel), and 72 h (right panel) post-infection. Cell viability is shown as a percentage of ATP content in mock-infected cells. Error bars indicate standard deviation (SD). * *p* < 0.05; ** *p* < 0.01; *** *p* < 0.001 as determined for rsT1L vs. rsT3D by Student’s *t*-test. @, *p* < 0.05; @@, *p* < 0.01; @@@, *p* < 0.001 as determined for T1L vs. T3D by Student’s *t*-test. The data shown is compiled from three independent experiments.

To assess the oncolytic capacity of recombinant reoviruses, we infected a panel of NSCLC cell lines (Table 1) with recombinant (rsT1L or rsT3D) or non-recombinant (T1L or T3D) reoviruses. The panel included two large cell carcinoma lines (H661 and H1299) and three adenocarcinoma lines (H1437, H1563, and H1975) (Table 1). Each cell line was mock infected or infected with rsT1L, rsT3D, T1L, or T3D at multiplicities of infection (MOIs) of 10, 100, or 1000 plaque forming units (PFU)/cell and ATP content was measured at 24, 48, and 72 h post-infection as an indicator of cell viability (Figure 1). We observed a dramatic difference in cell viability between rsT1L and rsT3D in the two large cell carcinoma cell lines (Figure 1A,B). In H661 cells, each dose of rsT1L induced more cell death than rsT3D at 48 and 72 h. The peak loss of cell viability following rsT3D infection was approximately 40% of control, whereas rsT1L reduced cell viability by greater than 95%. In H1299 cells, viability of rsT3D-infected cells never dropped below 60% of control, whereas rsT1L caused almost complete cell death at MOIs of 100 and 1000 PFU/cell by 72 h. In contrast to the large cell carcinoma cell lines, rsT1L and rsT3D induced comparable dose- and time-dependent cell killing in the adenocarcinoma cell lines (Figure 1C–E). Importantly, cell death induced in each NSCLC line by non-recombinant T1L and T3D phenocopied the cell killing induced by rsT1L and rsT3D, including enhanced killing of the two large cell carcinoma cell lines (H661 and H1299) by T1L compared to T3D (Figure 1A,B). These results indicate that the cell killing capacity of reovirus generated by plasmid-based reverse genetics does not differ from that of native, non-recombinant strains. These data also revealed that the two large cell carcinoma lines were more resistant to killing by rsT3D than rsT1L. These findings suggest that T1 reoviruses may be more effective oncolytics than T3 reoviruses in certain tumor types, including large cell carcinomas.

### 2.2. Reovirus Infectivity in NSCLC Cell Lines

**Figure 2:**
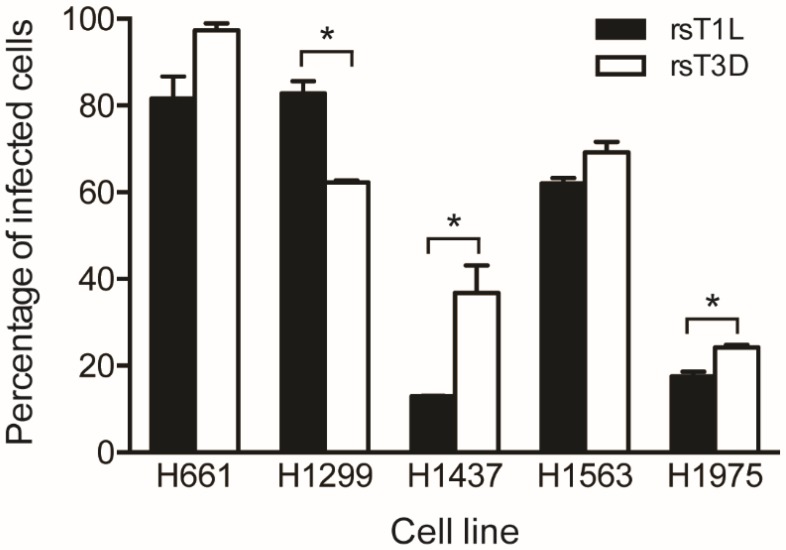
Reoviruses infect NSCLC cell lines. H661, H1299, H1437, H1563 and H1975 cells were infected with rsT1L or rsT3D at an MOI of 100 PFU/cell. At 24 h post-inoculation, reovirus-infected cells were detected by indirect immunofluorescence using reovirus-specific polyclonal antiserum. Nuclei were stained with DAPI. Results are expressed as the mean percentage of infected cells for duplicate samples. Error bars indicate SD. * *p* < 0.05 difference between rsT1L and rsT3D as determined by Student’s *t*-test. The data presented is compiled from two independent experiments.

We performed a fluorescent focus assay to assess reovirus infectivity in the NSCLC cell panel (Figure 2). Each cell line was infected with rsT1L or rsT3D at an MOI of 100 PFU/cell, which was the median viral dose tested in the cell killing experiments. At 18 h post-infection, the cells were fixed and stained using reovirus-specific polyclonal antiserum and DAPI. A higher percentage of H661, H1473, H1563, and H1975 cells were infected by rsT3D compared to rsT1L. H1299 cells were the only cell line that rsT1L infected to a greater degree than rsT3D (~80% vs. ~60%). The largest difference was observed in H1437 cells, where rsT3D infected an approximately 2-fold greater number of cells than rsT1L. In H661, H1299, and H1563 lines, the majority of cells were infected by both reovirus strains (>60%). In contrast, a low percentage (<40%) of H1437 and H1975 cells were infected by rsT1L or rsT3D. These findings indicate that each lung cancer cell lines tested is susceptible to infection by recombinant reoviruses. It is of note that although a higher percentage of H661 cells were infected with rsT3D than rsT1L, rsT1L induced markedly more cell death in H661 cells than rsT3D. Thus, it is unlikely that the strain-specific difference in killing of H661 cells is due to differential infectivity between the two viruses.

**Figure 3:**
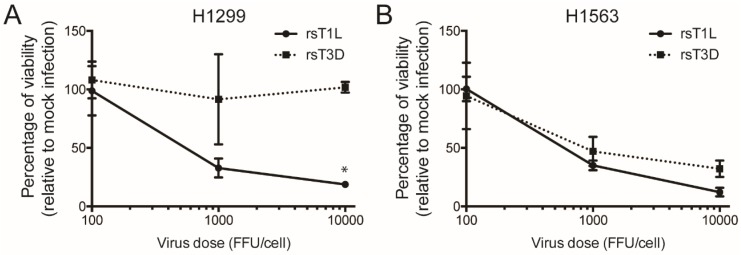
Differences in infectivity do not underlie reovirus strain-specific killing of H1299 cells. (**A**) H1299 or (**B**) H1563 cells were infected with rsT1L or rsT3D at MOIs of 100, 1000, or 10,000 fluorescent focus units (FFU)/cell. FFU titers determined on H1299 or H1563 cells were used to calculate the MOIs for infection. Cellular ATP content was measured at 72 h post-infection. Cell viability is shown as a percentage of ATP content in mock-infected cells. Error bars indicate SD. ** *p* < 0.001 as determined by Student’s *t*-test. The data presented is compiled from two independent experiments.

Although rsT1L and rsT3D infectivity did not differ in H661 cells, rsT1L infected H1299 cells more efficiently than rsT3D. A greater level of infection by rsT1L could result in more killing of H1299 cells than rsT3D. To test whether the disparity in infectivity caused strain-specific differences in killing of H1299 cells, we performed cell viability assays in which the infectious dose of rsT1L and rsT3D was normalized based on fluorescent focus unit (FFU) titers that were determined on H1299 cells. We infected H1299 cells with 10,000, 1000, or 100 FFU/cell and measured ATP at 72 h post-infection as an indicator of cell viability (Figure 3). For reovirus, the FFU:PFU ratio is approximately 10:1 [36]. Thus, the FFU doses tested approximate the MOI range used in experiments initiated using PFUs. We found that when infection was normalized by infectivity, rsT1L retained the capacity to kill H1299 cells to a greater degree than rsT3D (Figure 3A). H1563 cells, which were equally susceptible to rsT1L and rsT3D infection, were killed to comparable levels by rsT1L and T3D when infection was initiated based on normalize FFU titers that were determined on H1563 cells (Figure 3B). This result indicates that differences in virus infectivity do not lead to strain-specific differences in killing of H1299 cells.

### 2.3. rsT1L and rsT3D Gene Expression and Replication in NSCLC Cell Lines

**Figure 4:**
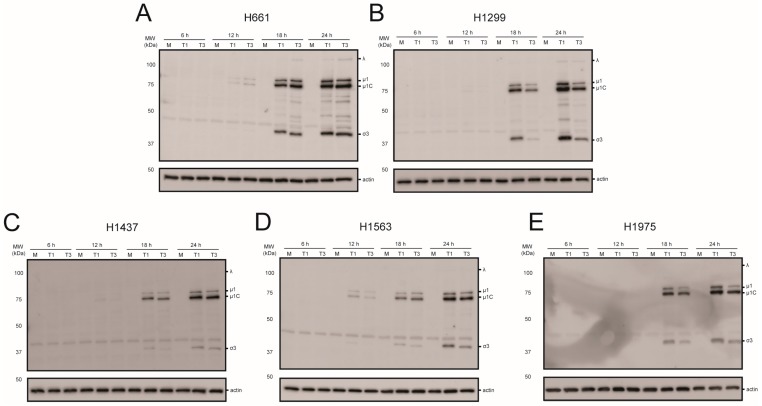
Reovirus protein synthesis in NSCLC cell lines. (**A**) H661; (**B**) H1299; (**C**) H1437; (**D**) H1563; and (**E**) H1975 cells were mock infected or infected with rsT1L or rsT3D at an MOI of 10 PFU/cell. Whole cell lysates prepared at 6, 12, 18, or 24 h post-infection were analyzed by SDS-PAGE and immunoblotted using reovirus-specific antiserum (top) or β-actin (bottom). Reovirus proteins are indicated to the right of the top panel. The images are representative of the results from two independent experiments.

To assess viral protein production in the NSCLC cell panel, each cell line was mock infected or infected with rsT1L or rsT3D at an MOI of 10 PFU/cell (Figure 4). Whole cell lysates prepared at 6, 12, 18, or 24 h post-infection were separated by SDS-PAGE and immunoblotted with reovirus-specific polyclonal antiserum. Reovirus proteins were detected in each cell line by 18 h. In H1299 and H1975 cells rsT1L produced modestly higher viral protein levels than rsT3D. In H1299 cells, rsT1L produced 3.8- and 3.6-fold more μ1 than rsT3D at 18 h and 24 h, respectively. Approximately 22-fold more σ3 was detected in rsT1L-infected cells relative to rsT3D at 18 h, but the difference decreased to 3.7-fold at 24 h. In H1975 cells, rsT1L produced 3- and 3.8-fold more μ1 at 18 h and 24 h, respectively. The differences in σ3 were 3.7-fold at 18 h and 2-fold at 24 h. For H1299, the difference could result from an increased number of cells becoming infected by rsT1L compared to rsT3D. However, no difference in reovirus gene expression was observed between rsT1L and rsT3D in H661, H1437, or H1563 cells. Together, these results demonstrate that recombinant reoviruses efficiently express viral proteins in NSCLC cell lines.

**Figure 5:**
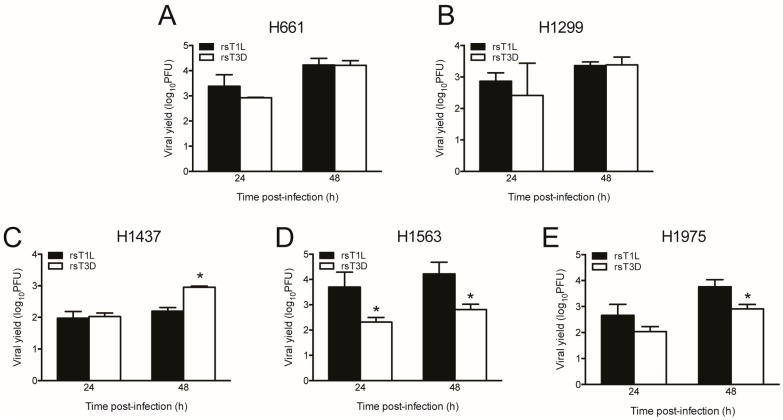
T1 and T3 reoviruses replicate in NSCLC cell lines. (**A**) H661; (**B**) H1299; (**C**) H1437; (**D**) H1563; and (**E**) H1975 cells were infected with rsT1L or rsT3D at an MOI of 1 PFU/cell. Viral titers were determined in cell lysates at 0, 24, and 48 h post-infection by plaque assay on L929 cells. Results are expressed as the mean viral yield at 24 and 48 h for triplicate samples. Error bars indicate SD. * *p* < 0.05 difference between rsT1L and rsT3D as determined by Student’s *t*-test. The data presented are representative of the results from two independent experiments.

To assess viral replication of the NSCLC cells, we infected each cell line with rsT1L or rsT3D at an MOI of 1 PFU/cell and quantified viral titers at 0, 24, and 48 h post-infection (Figure 5). We used a lower multiplicity infection for assessing viral replication than cell killing (1 PFU/cell versus 10–1000 PFU/cell) to provide a larger dynamic range in which to measure viral yields. We found that rsT1L and rsT3D replicated in each lung cancer cell line, with progeny yields ranging from 100- to 10,000-fold increase over input. We found that rsT1L and rsT3D produced comparable yields in H661 and H1299 cells. This result indicates that differences viral in replication do not account for differential killing of H661 and H1299 cells by rsT1L and rsT3D. H1437 cells produced the lowest viral yields of the cell lines tested. Yields of rsT1L and rsT3D were equivalent in H1437 cells at 24 h. However, rsT3D produced higher progeny yields than rsT1L at 48 h. Although rsT1L and rsT3D replicated in H1563 and H1975, rsT1L produced higher progeny yields than rsT3D in these cell lines. These results indicate that recombinant reoviruses replicate efficiently in all of the NSCLC cell lines tested.

### 2.4. Recombinant Reoviruses Kill NSCLC Cell Lines by a Caspase-Independent Mechanism

**Figure 6:**
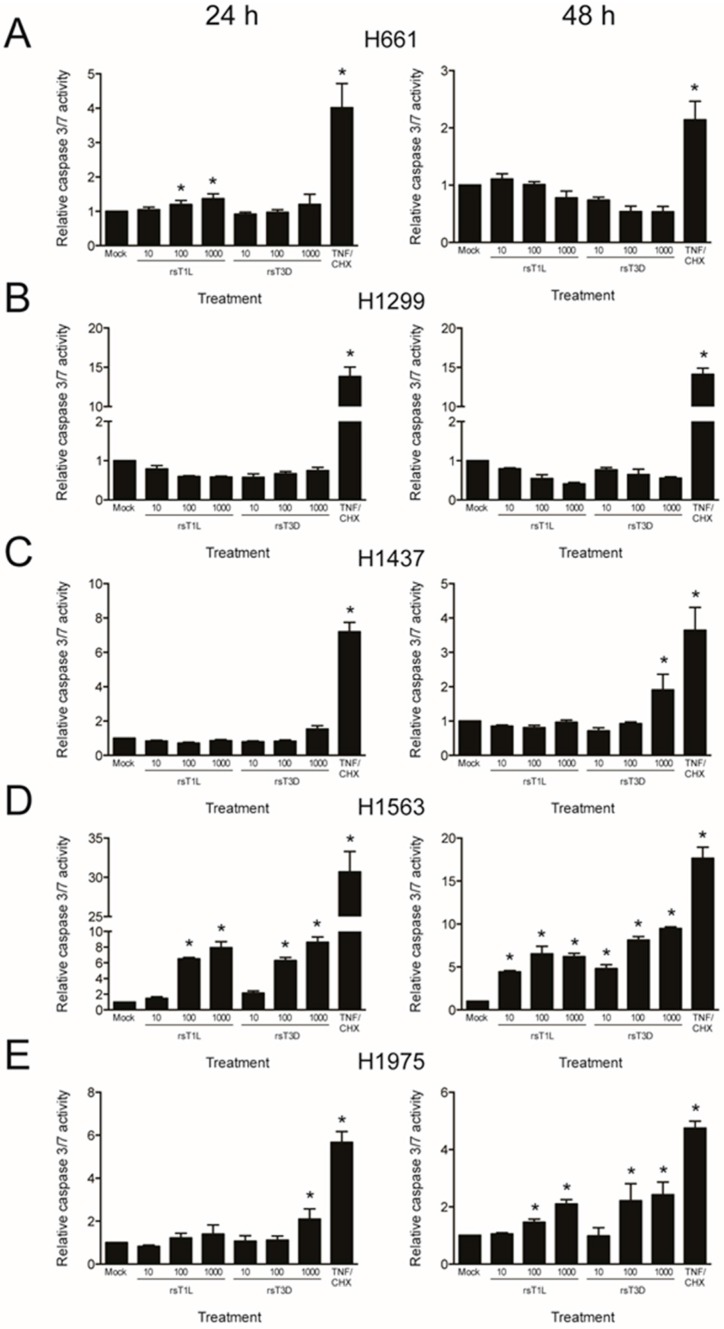
Caspase-3/7 activation in NSCLC cell lines during reovirus infection. (**A**) H661; (**B**) H1299; (**C**) H1437; (**D**) H1563; and (**E**) H1975 cells were mock infected or infected with rsT1L or rsT3D at an MOI of 100 PFU/cell. Caspase-3/7 activity was quantified at 24 h (left panel) or 48 h (right panel) post-infection. As a control, each cell line was treated with tumor necrosis factor-α (TNFα), 5 ng/mL) and cycloheximide (CHX, 25 μg/mL) 12 h prior to each time point. Results are presented as the mean caspase-3/7 activity relative to mock infection. Error bars indicate SD. * *p* < 0.05 relative to mock infection as determined by Student’s *t*-test. The data presented is representative of three independent experiments.

**Figure 7:**
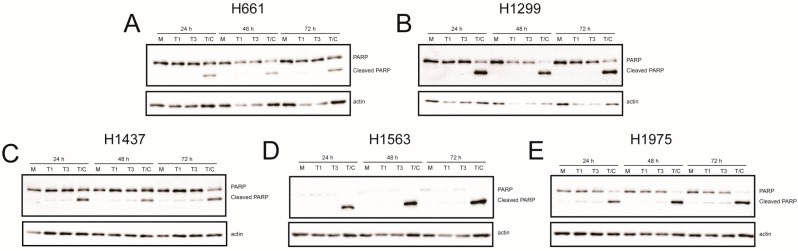
Reovirus-induced poly-ADP ribose polymerase (PARP) cleavage in NSCLC cell lines. (**A**) H661; (**B**) H1299; (**C**) H1437; (**D**) H1563; and (**E**) H1975 cells were mock infected (M) or infected with rsT1L (T1) or rsT3D (T3) at an MOI of 100 PFU/cell. Each cell line was treated with TNFα (5 ng/mL) and CHX (25 μg/mL) (T/C) 6 h prior to the 24 h time point. Whole cell lysates prepared at 24, 48, or 72 h post-infection were analyzed by SDS-PAGE and immunoblotted using a PARP-specific monoclonal antibody (top) and β-actin (bottom). Full-length and cleaved PARP proteins are indicated to the right of the upper panel. The images are representative of two independent experiments.

In many cell types, including L929 and HeLa cells, T3 reoviruses induce higher levels of apoptosis than T1 reoviruses [25]. To determine whether differences in apoptosis underlie differential cell killing by rsT1L and rsT3D, we assessed caspase-3/7 activity (Figure 6) and poly-ADP ribose polymerase (PARP) cleavage (Figure 7) in each NSCLC cell line following rsT1L and rsT3D infection. Tumor necrosis factor-α/cycloheximide (TNF-α/CHX) induced caspase-3/7 and PARP cleavage in each NSCLC cell line, indicating that apoptotic responses are functional in the cell panel. Infection with rsT1L or rsT3D did not elicit caspase-3/7 activity (Figure 6A,B) or PARP cleavage (Figure 7A,B) in the two large cell carcinoma lines (H661 and H1299). We detected modest caspase-3/7 activity in H1437 cells following infection with the highest multiplicity of rsT3D (Figure 6C). However, minimal PARP cleavage was observed in this cell line following reovirus infection (Figure 7C). In H1563 cells, rsT1L and rsT3D induced caspase-3/7 activity in a dose-dependent manner at 24 and 48 h (Figure 6D). However, rsT1L and rsT3D did not induce PARP cleavage in H1563 cells (Figure 7D). In H1975 cells, rsT1L and rsT3D elicited caspase-3/7 activity at 48 h (Figure 6E), but minimal PARP cleavage at all of the time points tested (Figure 7E).

**Figure 8:**
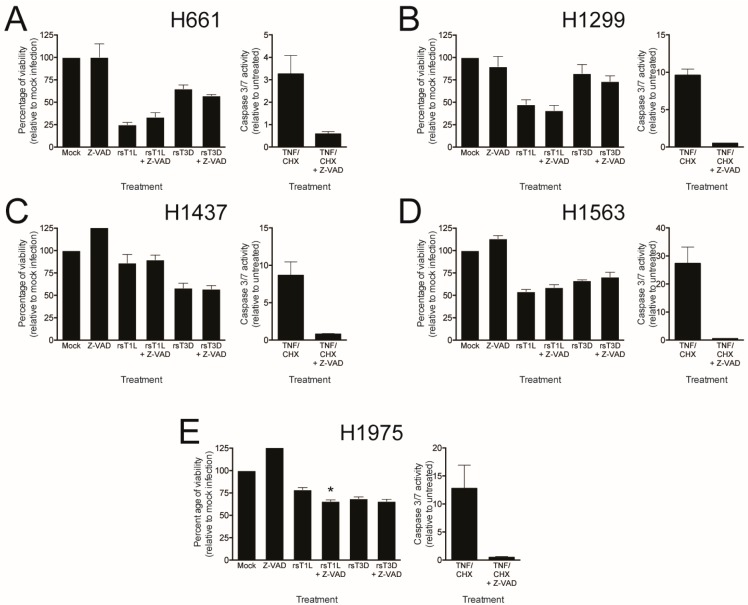
Reovirus-induced killing in NSCLC cell lines is caspase-independent. (**A**) H661; (**B**) H1299; (**C**) H1437; (**D**) H1563; and (**E**) H1975 cells were mock infected or infected with rsT1L or rsT3D at an MOI of 100 PFU/cell in the absence or presence of 25 μM Z-VAD. Cellular ATP content was quantified at 48 h post-infection. Cell viability is shown as a percentage of ATP content in mock-infected cells (left). Each cell line was treated with 25 μM Z-VAD for 1 h prior to mock treatment or treatment with TNFα (5 ng/mL) and CHX (25 μg/mL). Caspase-3/7 activity was quantified at 24 h and results are presented as the mean caspase-3/7 activity relative to mock infection (right). Error bars indicate SD. * *p* < 0.05 relative to mock infection as determined by Student’s *t*-test. The data presented is representative of two independent experiments.

To directly test whether caspases are required for reovirus killing of NSCLC cells, we assessed the effect of the pan-caspase inhibitor Z-VAD-FMK (Z-VAD) on reovirus cell killing (Figure 8). Each lung cancer cell line was infected with rsT1L or rsT3D at an MOI of 100 PFU/cell in the absence or presence of 25 μM Z-VAD and ATP content was measured at 48 h post-infection as an indicator of cell viability. TNF/CHX-induced caspase-3/7 activity was completely inhibited in each cell line by both Z-VAD concentrations, indicating that the dose of Z-VAD tested was functional. As before, rsT1L killed H661 and H1299 cells to a greater extent than rsT3D (Figure 8A,B). Similarly, rsT1L and rsT3D induced comparable levels of cell death in H1437, H1563, and H1975 cells (Figure 8C–E). We found that Z-VAD had little effect on killing by rsT1L or rsT3D in any of the NSCLC cell lines. Together, these results indicate that caspase activity is dispensable for reovirus-induced cell death in the NSCLC cell lines tested. These findings also suggest that strain-specific differences in apoptosis induction are not likely the underlying reason for the disparity in cell killing between rsT1L and rsT3D in H661 and H1299 cells.

### 2.5. Reovirus Gene Segments L2, L3, and M1 Correlate with Strain-Specific Differences in Cell Killing

**Figure 9:**
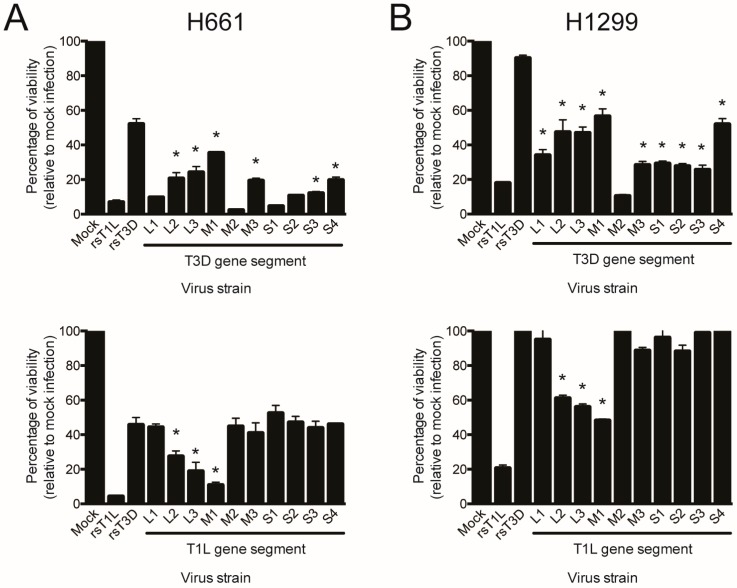
Reovirus gene segments L2, L3, and M1 mediate serotype-specific differences in killing of large cell carcinoma cell lines. (**A**) H661 or (**B**) H1299 cells were mock infected or infected with rsT1L, rsT3D, T3D single-gene reassortant viruses (upper panel), or T1L single-gene reassortant viruses (lower panel) at an MOI of 100 PFU/cell. Cellular ATP content was quantified at 48 h post-infection. Cell viability is shown as a percentage of ATP content in mock-infected cells. Error bars indicate SD. * *p* < 0.05 relative to mock infection as determined by Student’s *t*-test. The data presented is representative of two independent experiments.

To identify reovirus gene segments responsible for differential killing of the two large cell carcinoma lines by rsT1L and rsT3D, we used reverse genetics to engineer reciprocal panels of T1L × T3D single-gene reassortant viruses. We generated a complete set of 20 single-gene reassortant viruses in which all ten gene segments were individually replaced in rsT1L or rsT3D genetic backgrounds. We infected H661 and H1299 cells with rsT1L, rsT3D, or the reassortant viruses at an MOI of 100 PFU/cell and measured ATP content at 72 h post-infection as an indicator of cell viability (Figure 9). In both cell lines, the parental viruses retained the serotype-specific differences in cell killing described above (Figure 1). No single gene from T3D reduced the cell killing capacity of rsT1L to levels observed for rsT3D in either cell line. In H661 cells, replacement of T1L L2, L3, M1, M3, S3, or S4 gene with the T3D allele decreased killing by viruses with an otherwise T1L genetic background (Figure 9A, upper panel). In H1299 cells, all of the individual T3D gene replacements except for M2 decreased the cell killing capacity of the virus (Figure 9B, upper panel). In both H661 and H1299 cells, T1L genes L2, L3, and M1 enhanced cell killing of an otherwise T3D virus, although not to the level of rsT1L (Figure 9A,B, lower panels). These findings indicate that the L2, L3, and M1 gene segments have the highest correlation with differences between rsT1L- and rsT3D-mediated cell killing in H661 and H1299 cells.

**Figure 10:**
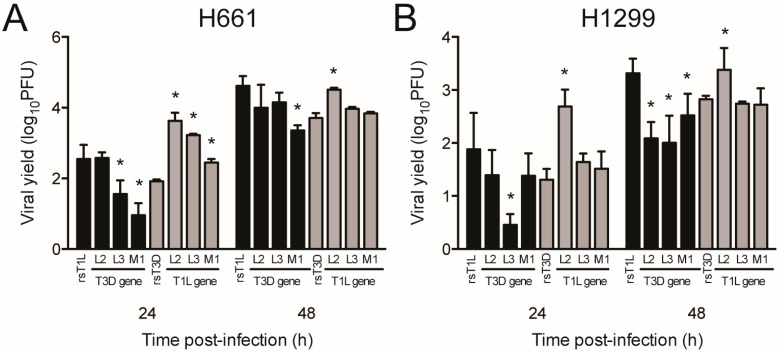
Replication of single-gene reassortant viruses in large cell carcinoma cell lines. (**A**) H661 or (**B**) H1299 cells were infected with rsT1L, rsT3D, or the indicated reassortant viruses at an MOI of 1 PFU/cell. Viral titers were determined in cell lysates at 0, 24, and 48 h post-infection by plaque assay on L929 cells. Results are expressed as the mean viral yield for triplicate samples at 24 h (right) and 48 h (left) post-infection. Error bars indicate SD. * *p* < 0.05 difference between rsT1L and rsT3D as determined by Student’s *t*-test. The data presented is representative of two independent experiments.

We next assessed replication of L2, L3, and M1 single-gene reassortment viruses in H661 and H1299 cells (Figure 10). We focused on L2, L3, and M1 because those genes exhibited the highest correlation with differences in cell killing in the reassortant panel. In H661 cells, rsT1L/T3D-L3 and rsT1L/T3D-M1 produced lower yields at 24 h relative to rsT1L. However, only rsT1L/T3D-M1 was reduced significantly at 48 h. In H1299 cells, only replication of rsT1L/T3D-L3 was substantially lower than rsT1L at 24 h. However, yields for all three T3D single gene replacements (rsT1L/T3D-L2, rsT1L/T3D-L3, and rsT1L/T3D-M1) were at least 10-fold reduced compared to wild type rsT1L at 48 h. In the T3D genetic background, only the virus with the T1L L2 gene replicated to higher levels than rsT3D at 48 h in both H661 and H1299 cells. These findings indicate that reovirus replication in the large cell carcinoma cell lines is influenced by the genetic complement of the virus.

## 3. Discussion

Our results indicate that recombinant reoviruses effectively kill NSCLC cell lines (Table 2). Importantly, cell killing by non-recombinant reoviruses was indistinguishable from cell death induced by recombinant strains, indicating that there are not significant differences in cell death induction between recombinant and native reoviruses. These results suggest that use of recombinant reoviruses to treat cancer can be as effective as the native strains. We also found that rsT1L killed the large cell carcinoma cell lines tested to a greater extent than rsT3D, suggesting that rsT1L or T1-based vectors may be more effective oncolytics than T3-based viruses in certain tumor types. A similar observation was made in a mouse mammary tumor line, where T1L induced more cell death than T3D, indicating that differential susceptibility of cancer cells to reovirus strains is not limited to the cells tested in this study [37].

It remains to be determined why the large cell carcinoma lines (H661 and H1299) differed in susceptibility to reovirus killing (Figure 1). One possibility is that H661 and H1229 cells harbor mutations in cancer-related genes or pathways that differ from those in the adenocarcinoma lines (H1437, H1563, and H1975). H1299 cells contain a mutation in the NRAS gene. H661 cells harbor mutations in the genes for CDKN2A, SMARCA4, and TP53. All three adenocarcinoma cell lines contain mutations in the CDKN2A gene, H1437 and H1975 cells contain mutations in the TP53 gene, and H1975 cells have mutations in the EGFR and PIK3CA genes. It is possible that mutations in other cancer-related genes associate with strain-specific differences in cell killing. However, based on the current information available, no common genetic marker correlates with differences in cell death induction between rsT1L and rsT3D in H661 and H1299 cells. A second possibility is whether the cell lines derive from primary or metastatic tumors (Table 1). Large cell carcinoma cell lines H661 and H1299 were isolated from metastatic growths, whereas adenocarcinoma cell lines H1563 and H1975 derive from a primary tumor mass. However, H1437 cells were generated from a lung metastasis. Moreover, data from mouse models and clinical trials indicate that T3D (Reolysin) can target and kill metastases in mouse models and humans [22,38,39]. Thus, whether cell lines derive from primary versus metastatic tumors is unlikely to underlie strain-specific differences in cell killing. Third, differential susceptibility to reovirus-induced cell death could reflect properties of the cell type of origin. Large cell carcinomas are undifferentiated tumors that originate from lung transformed epithelial cells [40], whereas adenocarcinomas derive from epithelial glands or ducts [41]. Although we found differential susceptibility to rsT3D between large cell carcinoma and adenocarcinoma cell lines, a previous study found that different adenocarcinoma and large cell carcinoma cell lines were equivalently susceptible to killing by Reolysin [42]. However, the same study identified bronchiolar carcinoma and lung squamous cell carcinoma cell lines that were Reolysin resistant [42]. Variable resistance to reovirus is observed in other cancer cell lines, including head and neck squamous cell carcinoma cell lines and pancreatic cancer cell lines [43,44]. It is common for human cancers to vary in numerous properties, such as growth rate, drug sensitivity, and invasiveness [45]. The same type of tumor can differ greatly between patients, and individual tumors are comprised of strikingly heterogeneous cell populations [45]. Tumor heterogeneity is a primary source of resistance to conventional chemotherapeutics [45]. Presumably, the mixed cell populations that comprise tumors will contain at least some cells that are resistant to reovirus. In clinical trials, some tumors respond well to reovirus therapy, whereas others were refractory to treatment [23]. Our results indicate that cancer cell lines resistant to one strain of reovirus may be susceptible to a different strain. In addition, serial passage in cancer cells can be used to select reovirus variants with enhanced cancer cell killing capacity [24]. Thus, many opportunities exist to use reverse genetics to expand the oncolytic range or potency of reovirus.

The difference in cell killing between rsT1L and rsT3D in H661 and H1299 cells also could result from distinct cell death mechanisms induced by T1 and T3 reoviruses. In cultured cells and in vivo, T3 reoviruses induce apoptosis, whereas T1 reoviruses induce minimal apoptosis and kills cells by an undefined mechanism [46,47,48,49]. Reolysin induces apoptosis in many different primary cancer cells and cancer cell lines, tumor xenografts in mice, and patients [44,50,51,52]. It is possible that H661 and H1299 cells have impaired apoptotic responses, which is a common feature of transformed cells. The inability to undergo apoptosis could prevent killing by rsT3D, but not rsT1L. However, we found that TNF-α/CHX induced apoptotic markers (caspase-3/7 activation (Figure 6) and PARP cleavage (Figure 7)) in H661 and H1299 cells, indicating that apoptotic responses remain intact in both cell lines. Moreover, minimal caspase-3/7 activity (Figure 6) and PARP cleavage (Figure 7) were observed following infection with either rsT1L or rsT3D in any of the cell lines tested. Furthermore, treatment with the broad-spectrum caspase inhibitor Z-VAD-FMK did not prevent killing by either reovirus strain in any of the cell lines tested (Figure 8). Taken together, these findings indicate that rsT1L and rsT3D can induce caspase-independent cell death in each NSCLC cell line used in this study. Caspase activity also is dispensable for killing of head and neck cancer cell lines [44]. In cancer patients, the mechanism of reovirus oncolysis is not well understood. In addition to apoptosis, reovirus also can induce autophagy and programmed necrosis [53,54]. It is possible that reovirus elicits multiple cell death pathways and that the cellular environment dictates the mechanism of cell killing.

We found that the L2, L3, and M1 genes associate with differences in killing between rsT1L and rsT3D in H661 and H1299 cells. The L2, L3, and M1 genes encode viral proteins λ2, λ1, and μ2, respectively [25]. Protein λ2 is an outer capsid component that forms pentamers at the 5-fold vertices of the reovirus virion [55,56]. A channel in the center of the λ2 pentamer serves as the insertion site for the σ1 protein [57]. Once σ1 dissociates during viral entry into target cells, the channel functions as the exit site for newly synthesized viral RNAs [58]. The λ2 protein also has guanylyltransferase [59] and methyltransferase [57] activity that is required for addition of 5′ cap to nascent reovirus transcripts. The λ1 protein is an inner capsid constituent that is present in low amounts in the virion (~120 copies) [60]. Protein λ1 has RNA helicase activity that may function in unwinding of genomic dsRNAs to initiate transcription [61,62,63]. The λ1 protein also has RTPase activity [63,64], which in combination with its helicase activity suggests that λ1 contributes to capping of reovirus mRNAs. The μ2 protein also is a low abundance inner capsid component (~24 copies) [60]. Protein μ2 is hypothesized to function as a polymerase cofactor that enhances reovirus replication in a number of cell lines by enhancing RNA synthesis [57]. However, the mechanism by which μ2 promotes reovirus RNA production is undefined. The μ2 protein also is implicated in serotype-specific differences in type 1-interferon induction and sensitivity [65,66,67]. It is not known whether λ1, λ2, and μ2 function independently or in concert to enhance rsT1L-mediated killing of H661 and H1299 cells. Although rsT1L and rsT3D replication in H661 and H1299 cells did not differ (Figure 5), replication of several of the single-gene reassortant viruses differed from the parental strains. We observed a general trend that insertion of T3D genes into a T1L genetic background decreased viral replication, whereas insertion of T1L genes into the T3D genetic background increased viral progeny production (Figure 10). Although differences in viral replication do not underlie differential cell killing by the parental viruses, variation in cell killing by single gene reassortant viruses may simply reflect differences in viral replication.

It is important to note that although rsT3D and Reolysin originally derive from laboratory strain T3D, each has a unique passage history and genetic differences between the two strains that could impact oncolytic efficacy. It remains to be determined whether H661 and H1299 cells are more resistant to Reolysin than rsT1L. Next generation sequencing of Reolysin stocks revealed 32 nucleotide changes between Reolysin and T3D reference sequences in Genbank, 18 of which resulted in protein coding changes [68]. A subsequent comparison of the Reolysin sequence and sequences of plasmid-encoded rsT3D gene segments identified 20 amino acid differences between the viruses [69]. These findings indicate that Reolysin and rsT3D are not highly divergent. Construction of the Reolysin polymorphisms in the rsT3D background will be important for mechanistic studies of reovirus oncolysis and an important step in moving recombinant reovirus to the clinic.

In this study, we assessed the oncolytic potential of recombinant reoviruses generated by plasmid-based reverse genetics. To our knowledge, this is the first characterization of cancer cell killing by recombinant reoviruses generated using plasmid-based reverse genetics [20]. Our findings suggest that in certain types of tumors, T1 reovirus-based oncolytics may be more effective than T3-based vectors. Importantly, the cell killing profiles of recombinant reoviruses mirrors that of the native strains, suggesting that recombinant viruses will be as effective as traditional strains with respect to their oncolytic capacity. This work will inform future efforts to improve the efficacy and safety of reovirus oncolytics.

## 4. Materials and Methods

### 4.1. Cell Lines

Lung cancer cell lines H661, H1299, H1437, H1563, and H1975 were obtained from the American Type Culture Collection (ATCC). All lung cancer cells were maintained at 37 °C in 5% CO2 in RPMI Medium 1640 (Gibco, Gaithersburg, MD, USA) supplemented to contain 10% fetal bovine serum (FBS) and 2mM l-glutamine. L929 cells were maintained in spinner culture at 37 °C in Joklik’s MEM (Sigma-Aldrich, St. Louis, MO, USA) supplemented to contain 5% FBS, 2 mM L-glutamine, 100 U/mL penicillin, 100 μg/mL streptomycin, and 25 ng/mL amphotericin B (Sigma-Aldrich).

### 4.2. Viruses

Non-recombinant reovirus strains T1L and T3D are laboratory stocks. Recombinant reoviruses were generated using plasmid-based reverse genetics as described [28,29]. Purified viral stocks were generated using second or third passage L929 cell lysates as described previously [70]. Viral particles were Vertrel-XF (Microcare, New Britain, CT, USA)-extracted from infected cell lysates, layered onto 1.2- to 1.4-g/cm^3^ CsCl gradients, and centrifuged at 29,000 rpm for 4 h. Bands corresponding to virions (1.36 g/cm^3^) [71] were collected and dialyzed in virion-storage buffer (150 mM NaCl, 15 mM MgCl_2_, 10 mM Tris-HCl (pH 7.4)). Plaque assay using L929 cells was used to determine viral titer [72].

### 4.3. Cell Viability Assay

Monolayers of cells in 96-well plates (1 × 10^4^ cells/well) were mock infected or adsorbed in triplicate with reovirus at room temperature (RT) for 1 h. The MOIs were calculated on PFU titers determined on L929 cells or FFU titers determined on H1299 or 1563 cells, as indicated. Monolayers were washed twice with phosphate-buffered saline (PBS) and incubated at 37 °C in completed media for various intervals. CellTiter-Glo (Promega, Madison, WI, USA) was used to assess cell viability [73,74,75]. Briefly, cells were cooled to RT, 100 μL of CellTiter-Glo was added to each well, and the plate was placed on a multi-purpose rotator for 2 min. Plates were incubated at RT for 10 min and luminescence was measured using a FluoStar Omega plate reader (BMG LabTech, Ortenberg, Germany). Where indicated, cells were treated with 25 μM Z-VAD-FMK (Sigma-Aldrich) or DMSO vehicle control for 1 h prior to infection. Following adsorption, the cells were overlaid with media containing DMSO or Z-VAD-FMK.

### 4.4. Virus Replication

Monolayers of cells in 24-well plates (1 × 10^5^ cells/well) were adsorbed with reovirus at a range of multiplicities at RT for 1 h. The monolayers were washed twice with PBS and incubated in completed media at 37 °C. At the indicated time points, cells were frozen and thawed twice, and viral titers in lysates were determined using plaque assay on L929 cells [72]. Viral yields were calculated using the following formula:

(1) log10yieldtx=log10(PFU/mL)tx−log10(PFU/mL)t0

where *tx* is the time post-infection.

### 4.5. Active Caspase-3/7 Assay

Monolayers of cells (1 × 10^4^ cells/well) in 96-well plates were mock infected or adsorbed in triplicate with reovirus at the indicated multiplicities at RT for 1 h. The monolayers were washed twice with PBS and incubated at 37 °C in completed media for various intervals. The cells were cooled to RT, 100 μL of Caspase-Glo 3/7 (Promega) was added to each well and the plates were incubated in the dark at RT for 1 h. Luminescence was measured using a FluoStar Omega plate reader (BMG LabTech).

### 4.6. Immunoblot Assay

Monolayers of cells (5 × 10^5^ cells/well) in 12-well plates were mock infected or adsorbed with reovirus at an MOI of 10 PFU/cell for 1h at RT. The monolayers were washed twice with PBS and incubated at 37 °C in completed media for various intervals. Cells were lysed with RIPA buffer (20 mM Tris-HCl, pH 7.5, 150 mM NaCl, 1 mM EDTA, 1% IGEPAL, 0.1% SDS, 0.1% deoxycholate) with 1% protease inhibitor cocktail (Sigma-Aldrich) and 1% phosphatase inhibitor cocktail (Sigma-Aldrich). Proteins were separated by SDS-PAGE and immunoblotted [49] using reovirus—(1:10,000 dilution), PARP—(Cell Signaling; 1:1000 dilution), or β-actin-specific (Sigma-Aldrich; 1:10,000 dilution) antibodies. Reovirus and β-actin antibodies were detected using goat-anti-rabbit Alexa-488 and goat-anti-mouse Alexa-546, respectively (1:5000 each). PARP and β-actin were detected using appropriate horseradish peroxidase-conjugated secondary antibodies (Jackson Immunoresearch, West Grove, PA, USA, 1:5000) and Super Signal West Pico reagent (Thermo-Fisher Scientific, Waltham, MA, USA). All blots were scanned using a ChemiDoc MP imaging system (Bio-Rad, Hercules, CA, USA). Imaging times were kept consistent for each cell line for replicate experiments. For viral protein quantitation, the band intensity for viral proteins was normalized to the intensity of the actin band in the corresponding land. The actin-normalized values for rsT1L and rsT3D were then compared. Viral protein quantitation was performed using Image Lab software, version 4.1 (Bio-Rad).

### 4.7. Indirect Immunofluorescence

Monolayers of cells in 96-well dishes (1 × 10^4^ cells/well) were adsorbed with reovirus at an MOI of 100 PFU/cell for 1h at RT. The monolayers were washed twice with PBS and incubated at 37 °C in completed media for 24 h. The media was removed and cells were fixed in methanol for 30 min at −20 °C. Fixed cells were incubated with PBS + 5% bovine serum albumin (BSA) for 15 min at RT followed by staining with reovirus-specific polyclonal antiserum (1:500 dilution in PBS + 0.5% Triton X-100) for 30 min at 37 °C. The cells were washed three times with PBS and incubated with Alexa-488-conjugated goat-anti-rabbit IgG (Life Technologies, Thermo-Fisher Scientific, 1:1000 dilution in PBS + 0.5% Triton X-100) and DAPI (1:1000 dilution) at 30 min at 37 °C. Cells were washed three times with PBS and visualized with an EVOS-FL Auto imaging system (Life Technologies, Thermo-Fisher Scientific). Infected cells were quantified by counting the number of reovirus antigen-positive cells along with the number of DAPI-stained nuclei (total cell number) in three separate fields of view in two independent experiments. The percentage of infected cells was calculated by dividing the number of reovirus antigen-positive cells by the number of DAPI-positive nuclei.

### 4.8. Statistical Analysis

Means for cell viability experiments were determined for three independent experiments, each of which contained three replicates. Differences were determined using random effects analysis of variance. Differences in cell killing between recombinant and non-recombinant viruses, cell infectivity, viral replication, and caspase-3/7 activity were determined using an unpaired Student’s *t*-test. Statistical analysis was performed using Prism software (GraphPad Software Inc., La Jolla, CA, USA). *p* values <0.05 were considered to be statistically significant.

## Figures and Tables

**Table 1:** Non-small cell lung cancer (NSCLC) cell lines used in this study.

Cell Line	Histology	Tumor Source
NCI-H661	Large cell carcinoma	Lymph node metastasis
NCI-H1299	Large cell carcinoma	Lymph node metastasis
NCI-H1437	Adenocarcinoma	Pleural effusion metastasis
NCI-H1563	Adenocarcinoma	Primary
NCI-H1975	Adenocarcinoma	Primary

**Table 2:** Summary of results. Cell killing, infectivity, replication, and apoptosis induction by recombinan strains (rs)T1L and rsT3D in each cell line was ranked on a graded scale. Cell killing was scored based on the percent loss of cell viability at 72 h at a multiplicity of infection (MOI) of 1000 plaque forming units (PFU)/cell (Figure 1) (+, less than 50%; ++, 50–80%; +++, 80–100%). Infectivity was scored based on the percentage of cells infected at 24 h (Figure 2) (+, less than 30%; ++, 30–80%; +++, 80–100%). Replication was scored based on viral progeny yields generated at 48h (Figure 5) (+, 0–2 log10; ++, 2–4 log10; +++, greater than 4 log10). Apoptosis induction was scored based on fold caspase-3/7 activation at 48 h at an MOI of 1000 PFU/cell (Figure 6) (−, no apoptosis induction; +, 1–2-fold; ++, 2–4-fold; +++, greater than 4-fold).

Cell Line	Cell Killing		Infectivity		Replication		Apoptosis Induction	
	rsT1L	rsT3D	rsT1L	rsT3D	rsT1L	rsT3D	rsT1L	rsT3D
H661	+++	++	+++	+++	+++	+++	−	−
H1299	+++	+	+++	++	++	++	−	−
H1437	++	++	+	++	+	++	−	+
H1563	+++	+++	++	++	+++	++	+++	+++
H1975	++	++	+	+	++	++	+	+

## References

[1] Russell S.J., Peng K.W., Bell J.C. (2012). Oncolytic virotherapy. Nat. Biotechnol..

[2] Miest T.S., Cattaneo R. (2014). New viruses for cancer therapy: Meeting clinical needs. Nat. Rev. Microbiol..

[3] Duncan M.R., Stanish S.M., Cox D.C. (1978). Differential sensitivity of normal and transformed human cells to reovirus infection. J. Virol..

[4] Coffey M.C., Strong J.E., Forsyth P.A., Lee P.W. (1998). Reovirus therapy of tumors with activated Ras pathway. Science.

[5] Hirasawa K., Nishikawa S.G., Norman K.L., Alain T., Kossakowska A., Lee P.W. (2002). Oncolytic reovirus against ovarian and colon cancer. Cancer Res..

[6] Norman K.L., Coffey M.C., Hirasawa K., Demetrick D.J., Nishikawa S.G., DiFrancesco L.M., Strong J.E., Lee P.W. (2002). Reovirus oncolysis of human breast cancer. Hum. Gene. Ther..

[7] Strong J.E., Coffey M.C., Tang D., Sabinin P., Lee P.W. (1998). The molecular basis of viral oncolysis: Usurpation of the Ras signaling pathway by reovirus. EMBO J..

[8] Wilcox M.E., Yang W., Senger D., Rewcastle N.B., Morris D.G., Brasher P.M., Shi Z.Q., Johnston R.N., Nishikawa S., Lee P.W. (2001). Reovirus as an oncolytic agent against experimental human malignant gliomas. J. Natl. Cancer Inst..

[9] Comins C., Heinemann L., Harrington K., Melcher A., De Bono J., Pandha H. (2008). Reovirus: Viral therapy for cancer “as nature intended”. Clin. Oncol..

[10] Comins C., Spicer J., Protheroe A., Roulstone V., Twigger K., White C.M., Vile R., Melcher A., Coffey M.C., Mettinger K.L. (2010). REO-10: A phase I study of intravenous reovirus and docetaxel in patients with advanced cancer. Clin. Cancer Res..

[11] Turnbull S., West E.J., Scott K.J., Appleton E., Melcher A., Ralph C. (2015). Evidence for Oncolytic Virotherapy: Where Have We Got to and Where Are We Going?. Viruses.

[12] Pol J., Kroemer G., Galluzzi L. (2016). First oncolytic virus approved for melanoma immunotherapy. Oncoimmunology.

[13] Liu B.L., Robinson M., Han Z.Q., Branston R.H., English C., Reay P., McGrath Y., Thomas S.K., Thornton M., Bullock P. (2003). ICP34.5 deleted herpes simplex virus with enhanced oncolytic, immune stimulating, and anti-tumour properties. Gene Ther..

[14] Vacchelli E., Eggermont A., Fridman W.H., Galon J., Zitvogel L., Kroemer G., Galluzzi L. (2013). Trial Watch: Immunostimulatory cytokines. Oncoimmunology.

[15] Stanford M.M., Barrett J.W., Nazarian S.H., Werden S., McFadden G. (2007). Oncolytic virotherapy synergism with signaling inhibitors: Rapamycin increases myxoma virus tropism for human tumor cells. J. Virol..

[16] Bischoff J.R., Kirn D.H., Williams A., Heise C., Horn S., Muna M., Ng L., Nye J.A., Sampson-Johannes A., Fattaey A. (1996). An adenovirus mutant that replicates selectively in p53-deficient human tumor cells. Science.

[17] Au G.G., Lindberg A.M., Barry R.D., Shafren D.R. (2005). Oncolysis of vascular malignant human melanoma tumors by Coxsackievirus A21. Int. J. Oncol..

[18] Grote D., Russell S.J., Cornu T.I., Cattaneo R., Vile R., Poland G.A., Fielding A.K. (2001). Live attenuated measles virus induces regression of human lymphoma xenografts in immunodeficient mice. Blood.

[19] Stojdl D.F., Lichty B., Knowles S., Marius R., Atkins H., Sonenberg N., Bell J.C. (2000). Exploiting tumor-specific defects in the interferon pathway with a previously unknown oncolytic virus. Nat. Med..

[20] Mohamed A., Johnston R.N., Shmulevitz M. (2015). Potential for Improving Potency and Specificity of Reovirus Oncolysis with Next-Generation Reovirus Variants. Viruses.

[21] Sabin A.B. (1959). Reoviruses: A new group of respiratory and enteric viruses formerly classified as ECHO type 10 is described. Science.

[22] Galanis E., Markovic S.N., Suman V.J., Nuovo G.J., Vile R.G., Kottke T.J., Nevala W.K., Thompson M.A., Lewis J.E., Rumilla K.M. (2012). Phase II trial of intravenous administration of Reolysin (Reovirus Serotype-3-dearing Strain) in patients with metastatic melanoma. Mol. Ther..

[23] Morris D.G., Feng X., DiFrancesco L.M., Fonseca K., Forsyth P.A., Paterson A.H., Coffey M.C., Thompson B. (2013). REO-001: A phase I trial of percutaneous intralesional administration of reovirus type 3 dearing (Reolysin) in patients with advanced solid tumors. Investig. New Drugs.

[24] Shmulevitz M., Gujar S.A., Ahn D.G., Mohamed A., Lee P.W. (2012). Reovirus variants with mutations in genome segments S1 and L2 exhibit enhanced virion infectivity and superior oncolysis. J. Virol..

[25] Dermody T.S., Parker J.S.L., Sherry B., Knipe D.M., Howley P.M. (2013). Orthoreovirus. Fields Virology.

[26] Greaves M., Maley C.C. (2012). Clonal evolution in cancer. Nature.

[27] Meacham C.E., Morrison S.J. (2013). Tumour heterogeneity and cancer cell plasticity. Nature.

[28] Kobayashi T., Antar A.A., Boehme K.W., Danthi P., Eby E.A., Guglielmi K.M., Holm G.H., Johnson E.M., Maginnis M.S., Naik S. (2007). A plasmid-based reverse genetics system for animal double-stranded RNA viruses. Cell Host Microbe.

[29] Kobayashi T., Ooms L.S., Ikizler M., Chappell J.D., Dermody T.S. (2010). An improved reverse genetics system for mammalian orthoreoviruses. Virology.

[30] Komoto S., Kawagishi T., Kobayashi T., Ikizler M., Iskarpatyoti J., Dermody T.S., Taniguchi K. (2014). A plasmid-based reverse genetics system for mammalian orthoreoviruses driven by a plasmid-encoded T7 RNA polymerase. J. Virol. Methods.

[31] Kanai Y., Komoto S., Kawagishi T., Nouda R., Nagasawa N., Onishi M., Matsuura Y., Taniguchi K., Kobayashi T. (2017). Entirely plasmid-based reverse genetics system for rotaviruses. Proc. Natl. Acad. Sci. USA.

[32] Siegel R.L., Miller K.D., Jemal A. (2017). Cancer Statistics, 2017. CA Cancer J. Clin..

[33] American Lung Association. http://www.lung.org.

[34] U.S. National Institutes of Health, National Cancer Institute SEER Cancer Statistics Review, 1975–2013. https://seer.cancer.gov/archive/csr/1975_2013/.

[35] American Cancer Society. http://www.cancer.org.

[36] Danthi P., Kobayashi T., Holm G.H., Hansberger M.W., Abel T.W., Dermody T.S. (2008). Reovirus apoptosis and virulence are regulated by host cell membrane penetration efficiency. J. Virol..

[37] Roner M.R., Mutsoli C. (2007). The use of monoreassortants and reverse genetics to map reovirus lysis of a ras-transformed cell line. J. Virol. Methods.

[38] Yang W.Q., Senger D.L., Lun X.Q., Muzik H., Shi Z.Q., Dyck R.H., Norman K., Brasher P.M., Rewcastle N.B., George D. (2004). Reovirus as an experimental therapeutic for brain and leptomeningeal metastases from breast cancer. Gene Ther..

[39] Kawaguchi K., Etoh T., Suzuki K., Mitui M.T., Nishizono A., Shiraishi N., Kitano S. (2010). Efficacy of oncolytic reovirus against human gastric cancer with peritoneal metastasis in experimental animal model. Int. J. Oncol..

[40] Travis W.D., Brambilla E., Noguchi M., Nicholson A.G., Geisinger K., Yatabe Y., Ishikawa Y., Wistuba I., Flieder D.B., Franklin W. (2013). Diagnosis of lung cancer in small biopsies and cytology: Implications of the 2011 International Association for the Study of Lung Cancer/American Thoracic Society/European Respiratory Society classification. Arch. Pathol. Lab. Med..

[41] Travis W.D., Brambilla E., Noguchi M., Nicholson A.G., Geisinger K., Yatabe Y., Ishikawa Y., Wistuba I., Flieder D.B., Franklin W. (2013). Diagnosis of lung adenocarcinoma in resected specimens: Implications of the 2011 International Association for the Study of Lung Cancer/American Thoracic Society/European Respiratory Society classification. Arch. Pathol. Lab. Med..

[42] Sei S., Mussio J.K., Yang Q.E., Nagashima K., Parchment R.E., Coffey M.C., Shoemaker R.H., Tomaszewski J.E. (2009). Synergistic antitumor activity of oncolytic reovirus and chemotherapeutic agents in non-small cell lung cancer cells. Mol. Cancer.

[43] Etoh T., Himeno Y., Matsumoto T., Aramaki M., Kawano K., Nishizono A., Kitano S. (2003). Oncolytic viral therapy for human pancreatic cancer cells by reovirus. Clin. Cancer Res..

[44] Twigger K., Roulstone V., Kyula J., Karapanagiotou E.M., Syrigos K.N., Morgan R., White C., Bhide S., Nuovo G., Coffey M. (2012). Reovirus exerts potent oncolytic effects in head and neck cancer cell lines that are independent of signalling in the EGFR pathway. BMC Cancer.

[45] Marusyk A., Polyak K. (2010). Tumor heterogeneity: Causes and consequences. Biochim. Biophys. Acta.

[46] Tyler K.L., Squier M.K., Brown A.L., Pike B., Willis D., Oberhaus S.M., Dermody T.S., Cohen J.J. (1996). Linkage between reovirus-induced apoptosis and inhibition of cellular DNA synthesis: Role of the S1 and M2 genes. J. Virol..

[47] Tyler K.L., Squier M.K., Rodgers S.E., Schneider B.E., Oberhaus S.M., Grdina T.A., Cohen J.J., Dermody T.S. (1995). Differences in the capacity of reovirus strains to induce apoptosis are determined by the viral attachment protein sigma 1. J. Virol..

[48] Fleeton M.N., Contractor N., Leon F., Wetzel J.D., Dermody T.S., Kelsall B.L. (2004). Peyer’s patch dendritic cells process viral antigen from apoptotic epithelial cells in the intestine of reovirus-infected mice. J. Exp. Med..

[49] Danthi P., Coffey C.M., Parker J.S., Abel T.W., Dermody T.S. (2008). Independent regulation of reovirus membrane penetration and apoptosis by the mu1 phi domain. PLoS Pathog..

[50] Thirukkumaran C.M., Nodwell M.J., Hirasawa K., Shi Z.Q., Diaz R., Luider J., Johnston R.N., Forsyth P.A., Magliocco A.M., Lee P. (2010). Oncolytic viral therapy for prostate cancer: Efficacy of reovirus as a biological therapeutic. Cancer Res..

[51] Kelly K.R., Espitia C.M., Mahalingam D., Oyajobi B.O., Coffey M., Giles F.J., Carew J.S., Nawrocki S.T. (2012). Reovirus therapy stimulates endoplasmic reticular stress, NOXA induction, and augments bortezomib-mediated apoptosis in multiple myeloma. Oncogene.

[52] Carew J.S., Espitia C.M., Zhao W., Kelly K.R., Coffey M., Freeman J.W., Nawrocki S.T. (2013). Reolysin is a novel reovirus-based agent that induces endoplasmic reticular stress-mediated apoptosis in pancreatic cancer. Cell Death Dis..

[53] Berger A.K., Danthi P. (2013). Reovirus activates a caspase-independent cell death pathway. mBio.

[54] Thirukkumaran C.M., Shi Z.Q., Luider J., Kopciuk K., Gao H., Bahlis N., Neri P., Pho M., Stewart D., Mansoor A. (2013). Reovirus modulates autophagy during oncolysis of multiple myeloma. Autophagy.

[55] Ralph S.J., Harvey J.D., Bellamy A.R. (1980). Subunit structure of the reovirus spike. J. Virol..

[56] White C.K., Zweerink H.J. (1976). Studies on the structure of reovirus cores: Selective removal of polypeptide lambda 2. Virology.

[57] Schiff L.A., Nibert M.L., Tyler K.L., Knipe D.M., Howley P.M. (2007). Orthoreoviruses and their replication. Fields Virology.

[58] Dryden K.A., Wang G., Yeager M., Nibert M.L., Coombs K.M., Furlong D.B., Fields B.N., Baker T.S. (1993). Early steps in reovirus infection are associated with dramatic changes in supramolecular structure and protein conformation: Analysis of virions and subviral particles by cryoelectron microscopy and image reconstruction. J. Cell Biol..

[59] Cleveland D.R., Zarbl H., Millward S. (1986). Reovirus guanylyltransferase is L2 gene product lambda 2. J. Virol..

[60] Coombs K.M. (1998). Stoichiometry of reovirus structural proteins in virus, ISVP, and core particles. Virology.

[61] Noble S., Nibert M.L. (1997). Core protein mu2 is a second determinant of nucleoside triphosphatase activities by reovirus cores. J. Virol..

[62] Noble S., Nibert M.L. (1997). Characterization of an ATPase activity in reovirus cores and its genetic association with core-shell protein lambda1. J. Virol..

[63] Bisaillon M., Bergeron J., Lemay G. (1997). Characterization of the nucleoside triphosphate phosphohydrolase and helicase activities of the reovirus lambda1 protein. J. Biol. Chem..

[64] Bisaillon M., Lemay G. (1997). Characterization of the reovirus lambda1 protein RNA 5′-triphosphatase activity. J. Biol. Chem..

[65] Sherry B., Baty C.J., Blum M.A. (1996). Reovirus-induced acute myocarditis in mice correlates with viral RNA synthesis rather than generation of infectious virus in cardiac myocytes. J. Virol..

[66] Sherry B., Torres J., Blum M.A. (1998). Reovirus induction of and sensitivity to beta interferon in cardiac myocyte cultures correlate with induction of myocarditis and are determined by viral core proteins. J. Virol..

[67] Zurney J., Kobayashi T., Holm G.H., Dermody T.S., Sherry B. (2009). Reovirus mu2 protein inhibits interferon signaling through a novel mechanism involving nuclear accumulation of interferon regulatory factor 9. J. Virol..

[68] Chakrabarty R., Tran H., Fortin Y., Yu Z., Shen S.H., Kolman J., Onions D., Voyer R., Hagerman A., Serl S. (2014). Evaluation of homogeneity and genetic stability of REOLYSIN (pelareorep) by complete genome sequencing of reovirus after large scale production. Appl. Microbiol. Biotechnol..

[69] Sandekian V., Lim D., Prud’homme P., Lemay G. (2013). Transient high level mammalian reovirus replication in a bat epithelial cell line occurs without cytopathic effect. Virus Res..

[70] Furlong D.B., Nibert M.L., Fields B.N. (1988). Sigma 1 protein of mammalian reoviruses extends from the surfaces of viral particles. J. Virol..

[71] Smith R.E., Zweerink H.J., Joklik W.K. (1969). Polypeptide components of virions, top component and cores of reovirus type 3. Virology.

[72] Virgin H.W.T., Bassel-Duby R., Fields B.N., Tyler K.L. (1988). Antibody protects against lethal infection with the neurally spreading reovirus type 3 (Dearing). J. Virol..

[73] Mainou B.A., Zamora P.F., Ashbrook A.W., Dorset D.C., Kim K.S., Dermody T.S. (2013). Reovirus cell entry requires functional microtubules. mBio.

[74] Berger A.K., Hiller B.E., Thete D., Snyder A.J., Perez E., Upton J.W., Danthi P. (2017). Viral RNA at Two Stages of Reovirus Infection Is Required for the Induction of Necroptosis. J. Virol..

[75] Van den Hengel S.K., Balvers R.K., Dautzenberg I.J., van den Wollenberg D.J., Kloezeman J.J., Lamfers M.L., Sillivis-Smit P.A., Hoeben R.C. (2013). Heterogeneous reovirus susceptibility in human glioblastoma stem-like cell cultures. Cancer Gene Ther..

